# Effectiveness of microperimetry in evaluating anti-vascular endothelial growth factor therapy for diabetic macular edema patients with relatively good vision: A retrospective observational study

**DOI:** 10.1097/MD.0000000000028404

**Published:** 2021-12-23

**Authors:** Masahiko Sugimoto, Yasuko Wakamatsu, Ryohei Miyata, Kumiko Kato, Hisashi Matsubara, Mineo Kondo

**Affiliations:** Department of Ophthalmology, Mie University Graduate School of Medicine, Tsu, Japan.

**Keywords:** anti-vascular endothelial growth factor treatment, diabetic macular edema, good visual acuity, microperimetry

## Abstract

No studies have evaluated the retinal sensitivity (RS) for diabetic macular edema (DME) patients with good vision. Therefore, this study aimed to determine the effectiveness of microperimetry in evaluating the effectiveness of anti-vascular endothelial growth factor (anti-VEGF) treatment for DME patients with relatively good vision.

Twenty-seven eyes of 27 patients (mean age, 61.3 ± 11.2 years) with DME and decimal best-corrected visual acuity (BCVA) ≥0.6 were studied. All patients received 3 consecutive monthly injections of intravitreal anti-VEGF agents. The BCVA, central subfield macular thickness (CMT), and RS were evaluated by microperimetry (MAIA) within the 10 degree of the foveal center. To determine significant differences between the values, we used paired *t* tests.

Patients were evaluated at baseline and 4 weeks after the third injection. The BCVA improved significantly from 0.18 ± 0.06 logarithm of the minimum angle of resolution (logMAR) units to 0.13 ± 0.13 logMAR units (*P* = .002; paired *t* test). The CMT decreased significantly from 464.3 ± 91.8 μm to 393.4 ± 129.0 μm (*P* = .005), and the RS also improved significantly from 21.8 ± 3.1 dB to 24.1 ± 2.8 dB at 4 weeks after treatment (*P* = .006). Among the patients with a decimal BCVA of 0.7 or better at baseline, there was no significant improvement in the BCVA (*P* = .28). However, the CMT decreased significantly from 479.5 ± 79.1 μm to 394.0 ± 99.8 μm at 4 weeks after treatment (*P* = .007). The RS also improved significantly from 22.0 ± 2.4 dB to 24.0 ± 3.1 dB at 4 weeks after treatment (*P* = .004).

Measuring RS by microperimetry is a good option for evaluating the effectiveness of anti-VEGF treatment for DME patients with a relatively good BCVA.

## 1 Introduction

Diabetic macular edema (DME) is the leading cause of blindness in working-age individuals.^[1,2]^ Although laser photocoagulation has been the criterion standard method of treating DME, it is difficult to obtain substantial improvements of the visual acuity (VA) by laser photocoagulation alone.

The vascular endothelial growth factor (VEGF) has recently been reported to enhance vascular proliferation and increase the hyperpermeability of retinal blood vessels. VEGF suppression has also been found to be effective in resolving DME.^[3]^ Many randomized controlled trials (RCTs) have demonstrated the effectiveness of anti-VEGF agents for DME, and anti-VEGF therapy has now become the first-line therapy for DME.^[4–8]^ At present, the goal of DME treatment has changed from the prevention of vision loss to vision improvement. However, previous RCTs excluded eyes with VA better than 20/32 (decimal best-corrected visual acuity [BCVA] of 0.63), and little is known about the effectiveness of anti-VEGF treatment in such eyes. Hence, the DRCR.net Protocol V was designed to investigate the usefulness of anti-VEGF treatment for DME eyes with good VA.^[9]^

For most RCTs, including Protocol V, the effectiveness of anti-VEGF treatment was evaluated by visual functions, such as the BCVA, or by anatomical parameters, such as the retinal thickness determined using optical coherence tomography (OCT). However, the BCVA tests can evaluate only 1 point in the central retina and do not provide information about the physiological status of the central retina.

Microperimetry (MP) is a relatively new method that can evaluate the physiological status of different sites within 10 degree of the foveal center.^[10]^ The MP findings allow clinicians to determine retinal damage at different sites and the effectiveness of treatment for various diseases.^[11–15]^ Although there have been several studies using MP on patients with DME,^[16–19]^ we are not aware of any studies that evaluated the retinal sensitivity (RS) of DME patients with good vision.

Therefore, the purpose of this study was to determine whether the central retinal function evaluated by MP is useful for evaluating the effectiveness of anti-VEGF treatment for DME patients with relatively good vision.

## 2 Methods

### 2.1 Ethics statements

The study procedures were approved by the Institutional Ethics Review Board of Mie University Hospital (#2628), and the protocol was registered at http://www.umin.ac.jp (UMIN ID 000012094). The procedures adhered to the tenets of the Declaration of Helsinki, and informed consent was obtained from all patients.

### 2.2 Study design and population

This retrospective observational study was performed in patients examined in the Department of Ophthalmology, Mie University Hospital from October 2013 to November 2018. Patients diagnosed with DME who received intravitreal anti-VEGF injections were recruited. The inclusion criteria were as follows: DME patients with treatment-naïe eyes, age ≥20 years, and a pretreatment decimal BCVA of ≥0.6 (20/33). The clinical diagnosis of DME was defined as central subfield macular thickness (CMT) >300 μm and based on clinical findings. The exclusion criteria were as follows: a history of ophthalmic surgery within 6 months before this study, history of pars plana vitrectomy, history of other DME treatment (ie, intravitreal or sub-Tenon steroid injection and macular laser photocoagulation within 3 months before this study), other ocular diseases (ie, severe PDR, drusen, glaucoma, ocular inflammation, retinal hemorrhage in the central macula, and severe media opacities); and history of thromboembolic events or systemic complications.

Bevacizumab (Avastin, Genentech, South San Francisco, CA), ranibizumab (Lucentis, Genentech), or aflibercept (Eylea;, Regeneron Pharmaceuticals, Tarrytown, NY) were used as anti-VEGF agents. Because bevacizumab was not approved in Japan, we obtained institutional review board approval for its use in our hospital (#702).

### 2.3 Evaluations, treatment, and data collection

Each patient underwent ophthalmological examinations before the first anti-VEGF treatment and 4 weeks after the 3 consecutive monthly anti-VEGF treatments. Measurements of BCVA, intraocular pressure, and slit-lamp examinations were performed. A subjective method of determining metamorphopsia was used during the slit-lamp examination with a 90-diopter lens, and the Watzke-Allen slit beam test (W/A test) was performed with a 100-μm length of slit beam focused on the macula. The patient was asked if the image of the slit beam appeared distorted or curved. If the patient's response was uncertain, we repeated the test until we obtained a defined response.^[20]^

The fundus examination was performed using indirect ophthalmoscopy. The severity of diabetic retinopathy (DR) was classified into 5 categories according to the International Clinical Diabetic Retinopathy Disease Severity Scale (DRSS): no DR, mild nonproliferative DR (mild NPDR), moderate NPDR, severe NPDR, and proliferative DR (PDR).^[21]^

Glycated hemoglobin A1c (HbA1c) (normal range, 4.6%–6.2%) was used to evaluate diabetes control. The estimated glomerular filtration rate (normal range: 60–120 mL/min/m^2^) was used to evaluate renal function.

#### 2.3.1 Intravitreal anti-vascular endothelial growth factor injections

With patients under local anesthesia, we injected .05 mL of intravitreal anti-VEGF agents with a 30-gauge needle under sterile conditions. All patients received 3 consecutive injections of the same agent monthly, as described previously in detail.^[22]^ We used a topical antibiotic (levofloxacin hydrate, 1.5% Cravit ophthalmic solution) for all patients after the injection.

#### 2.3.2 Microperimetry

RS was determined using the Macular Integrity Assessment (MAIA Center Vue, Padova, Italy), and the RS evaluation was performed as previously described.^[23]^ This instrument comprises an infrared fundus camera with software that can track eye movements automatically with respect to a reference frame obtained at the beginning of the measurements. Thus, the same area of the retina was measured during each examination. In a dark room, we performed the MAIA examination after 5 minutes of dark adaptation. We used the following parameters with a 4–2 threshold strategy. Under the 4-apostilb background luminance, a 1 degree diameter red circle was used as the fixation target. With a maximum luminance of 1000 apostilb, the stimulus size was set as Goldmann III with a dynamic range of 36 dB. The average RS obtained from a 37-stimuli grid covering the central 10 degree of the retina was defined as the average threshold of RS.

#### 2.3.3 Best-corrected visual acuity

We used a Landolt chart to measure the BCVA. The decimal BCVA was converted to the logarithm of the minimum angle of resolution (logMAR) units.

#### 2.3.4 Optical coherence tomography

The macular structure was determined using spectral domain-OCT (Spectralis^R^, Heidelberg Engineering Inc., Heidelberg, Germany). We used spectral domain-OCT (Spectralis^R^) to measure the CMT as previously described.^[22]^ From 25 horizontal lines that consisted of 1024 A-scans per line, we used the fast macula protocol for the evaluation. The thickness from the internal limiting membrane to the retinal pigment epithelium was defined as the CMT. With the bundled software, the macular thickness around the 1-mm and 3-mm circle of the center subfield was automatically calculated.

### 2.4 Statistical analyses

All data are presented as mean ± standard deviation. To determine significant differences between the values, we used paired *t* tests. *χ*^2^ tests were used to evaluate the differences in the DRSS score or the number of patients with metamorphopsia. A *P* value <.05 was defined as statistically significant. All calculations were performed using the Statcel 4 Statistical Program (Statcel; OMC, Saitama, Japan).

## 3 Results

### 3.1 Patient demographics

Twenty-seven eyes from 27 patients with DME (19 men and 8 women, mean age, 61.3 ± 11.2 years) were studied. Three eyes received bevacizumab, 18 eyes received ranibizumab, and 6 eyes received aflibercept. The DRSS score was mild NPDR in 2 eyes, moderate NPDR in 19 eyes, and severe NPDR in 6 eyes. The average HbA1c level was 7.6 ± 1.7% (range, 5.4–11.0), and the average estimated glomerular filtration rate was 77.1 ± 23.7 mL/min/m^2^ (range, 19.9–119.6). The average intraocular pressure at baseline was 17.4 ± 2.7 mmHg. Among the 27 eyes, 5 eyes had a history of cataract surgery, and 22 eyes did not. Twenty eyes received pan-retinal photocoagulation, and 7 eyes did not.

### 3.2 Changes in the variables in all eyes

Significant improvement was observed in the BCVA, CMT, and RS after three consecutive monthly treatments (Table 1). The BCVA significantly improved from 0.18 ± 0.06 (.74 decimal VA) before treatment to 0.13 ± 0.13 logMAR units (0.89 decimal VA) after treatment (*P* = .002; paired *t* test). The CMT of the central 1-mm circle decreased significantly from 464.3 ± 91.8 μm before treatment to 393.4 ± 129.0 μm after treatment (*P* = .005), and the CMT of the central 3-mm circle decreased significantly from 433.1 ± 61.0 μm before treatment to 392.7 ± 66.5 μm after treatment (*P* = .001). The RS threshold was 21.8 ± 3.1 dB before treatment, which significantly improved to 24.1 ± 2.8 dB after treatment (*P* = .006).

**Table 1:** Changes in the best-corrected visual acuity, central subfield macular thickness, and retinal sensitivity following antivascular endothelial growth factor therapy for all eyes.

		*CMT*, *μm*		
	*BCVA*	*1 mm*	*3 mm*	*RS*, *dB*
*Pre*	.18 ± .06	464.3 ± 91.8	433.1 ± 61.0	21.8 ± 3.1
*Post*	.13 ± .13^∗∗^	393.4 ± 129.0^∗∗^	392.7 ± 66.5^∗∗^	24.1 ± 2.8^∗∗^

Data are presented as the mean ± standard deviation. BCVA = best-corrected visual acuity, CMT = central subfield macular thickness, Post = posttreatment., Pre = pretreatment, RS = retinal sensitivity.

∗∗ *P* < .01, paired *t* test.

Among 27 eyes, 10 eyes had metamorphopsia as the chief complaint. Seven eyes showed significant improvement in metamorphopsia after treatment (*P* = .003; *χ*^2^ test). The DRSS score did not significantly improve after treatment (*P* = .10, *χ*^2^ test): mild NPDR, 4 eyes; moderate NPDR, 23 eyes; and severe NPDR or PDR, 0 eyes.

### 3.3 Changes in the variables of eyes with a decimal BCVA of 0.7 and better

Because the previous RCTs excluded eyes with a VA better than 20/32 (decimal BCVA, 0.63), we also analyzed eyes with a decimal BCVA of 0.7 and better (Table 2). Ten eyes of 10 patients (9 men and 1 woman) had a baseline decimal BCVA of 0.7 or better (range, .7–1.0 decimal VA). Six eyes received ranibizumab, and 4 eyes received aflibercept (mean age, 63.7 ± 9.9 years).

**Table 2:** Changes in the best-corrected visual acuity, central subfield macular thickness, and retina sensitivity following antivascular endothelial growth factor therapy for eyes with a decimal best-corrected visual acuity better than 0.7 (20/28).

		*CMT*, μm		
	*BCVA*	*1 mm*	*3 mm*	*RS*, *dB*
*Pre*	.12 ± .07	479.5 ± 79.1	438.8 ± 74.3	22.0 ± 2.4
*Post*	.09 ± .12	394.0 ± 99.8^∗∗^	382.4 ± 50.1^∗^	24.0 ± 3.1^∗∗^

Data are presented as the mean ± standard deviation. BCVA = best-corrected visual acuity, CMT = central subfield macular thickness, Post = posttreatment., Pre = pretreatment, RS = retinal sensitivity.

∗ *P* < .05.

∗∗ *P* < .01; paired *t* test.

The initial BCVA was 0.12 ± 0.07 logMAR units (.92 decimal VA) before treatment, and it improved but not significantly to 0.09 ± 0.12 logMAR units (1.05 decimal VA) after the three consecutive monthly treatments (paired *t* test, *P* = .28). The CMT of the central 1-mm circle decreased significantly from 479.5 ± 79.1 μm before treatment to 394.0 ± 99.8 μm after treatment (*P* = .007), and the CMT of the central 3-mm circle decreased significantly from 438.8 ± 61.0 μm before treatment to 382.4 ± 50.1 μm after treatment (*P* = .02). The RS threshold before treatment was 22.0 ± 2.4 dB, and it significantly improved to 24.0 ± 3.1 dB after treatment (*P* = .004).

### 3.4 Case presentation

**Figure 1:**
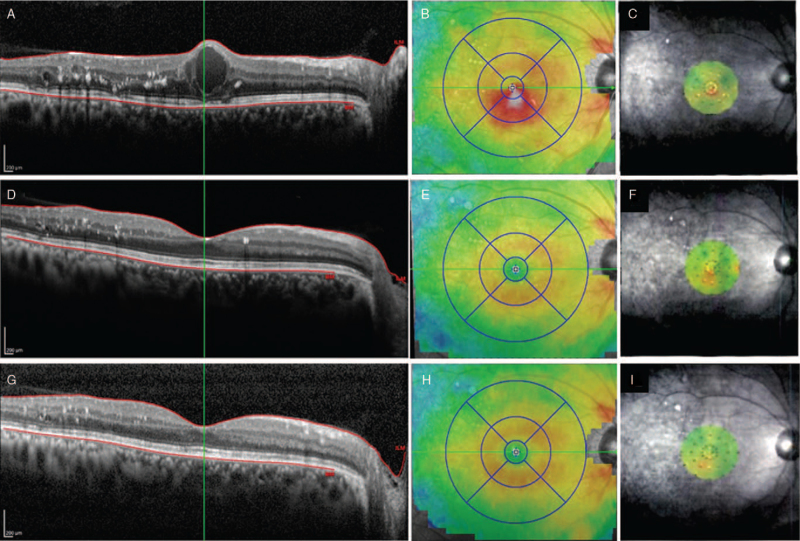
Case presentation. At the first visit, the BCVA of this patient had a decimal BCVA of 1.0 (20/20) with cystic DME (A). The CMT was 408 μm (B, shown as an OCT map), and the RS threshold was 24.4 dB (C, shown as a threshold map). Four weeks after three consecutive monthly injections of ranibizumab, the BCVA of the patient was a decimal BCVA of 1.0 (20/20) with a resolution of the cystic changes (D). The CMT of the patient was reduced to 265 μm (E), and the RS threshold was improved to 24.4 dB (F). Three months after treatment, the BCVA of the patient was 20/20 without recurrence of DME (G). The CMT of the patient improved to 260 μm (H), and the RS threshold of the patient improved to 26.3 dB (I). BCVA = best corrected visual acuity, CMT = central subfield macular thickness, DME = diabetic macular edema, OCT = optical coherence tomography, RS = retinal sensitivity.

The 79-year-old male patient had an initial decimal BCVA of 1.0 (Fig. 1A–I). Cystic DME with a CMT of 408 μm was observed. The chief complaint was metamorphopsia. After 3 consecutive monthly ranibizumab injections, the cyst disappeared, and the CMT improved to 265 μm. The BCVA remained at a decimal BCVA of 1.0, but the RS threshold before treatment was 24.4 dB, which significantly improved to 26.3 dB. His chief complaint of metamorphopsia disappeared after treatment.

## 4 Discussion

Our results showed that anti-VEGF treatment for DME patients with relatively good VA is effective, and MP is a good option to evaluate the effectiveness of this treatment. In addition, anti-VEGF treatment for these patients improved the degree of metamorphopsia due to DME.

Earlier RCTs did not include or evaluate the effect of anti-VEGF agents for DME patients with a decimal BCVA better than 0.66 (20/32) (Table 3).^[3,5–8,24]^ The DRCR.net Protocol V was designed to determine whether the anti-VEGF agents (eg, aflibercept) are effective for DME patients with a BCVA better than 20/25 (decimal BCVA, 0.8).^[9]^ The vision improvement was better for eyes treated with anti-VEGF agents than for eyes treated with laser photocoagulation at the 1-year observation. Similarly, Busch et al also reported on the 1-year outcomes of untreated or treated DME with various types of therapy in eyes with a VA better than 20/25 (decimal BCVA, 0.8) in real-world settings.^[25]^ However, the disadvantages of these studies were that the VA was the only parameter used to evaluate visual function. Although the VA is an important physiological parameter, it does not always reflect the comprehensive visual functions^[26]^ because it can measure the retinal function only in the central 1° of the visual field.^[27]^ Major symptoms of DME, such as metamorphopsia, aniseikonia, distortion, and blurring, are not fully reflected in the VA evaluation.^[28,29]^ Other visual function tests, for example, the Amsler chart, M-chart, preferential hyperacuity perimetry, contrast sensitivity, and electroretinography add detailed information of the visual function in macular diseases.^[30]^ These examinations provide different aspects of visual function, and MP has the advantage of assessing additional information in the central area of 10° around the fovea quantitatively.^[29,31,32]^ In addition, follow-up examinations have been useful to evaluate the effectiveness of treatments.^[33,34]^ Here, we showed improved of RS in DME patients with comparatively good VA, although the BCVA did not improve significantly for patients with a BCVA >0.7. Thus, we believe that MP is a good option for evaluating central retinal function.

**Table 3:** Eligible maximum visual acuity of various randomized controlled trials.

RCT	Eligible maximum VA (decimal)
RESOLVE (Massin et al, 2010)^[5]^	20/40 (.5)
RESTORE (Mitchell et al, 2011)^[6]^	20/32 (.6)
RISE/RIDE (Nguyen et al, 2012)^[7]^	20/40 (.5)
VIVID/VISTA (Brown et al, 2015)^[8]^	20/40 (.5)
Protocol I (Elman et al, 2015)^[3]^	20/32 (.6)
Protocol T (DRCR.Net 2015)^[24]^	20/32 (.6)

RCT = randomized controlled trial, VA = visual acuity.

Metamorphopsia is a major symptom in patients with macular diseases that affects the quality of visual function, and displacement of the outer segments of the photoreceptors is the cause of this symptom.^[35]^ In the diabetic retina, chronic hyperglycemia causes oxidative or inflammatory damage, resulting in the breakdown of the blood–retinal barrier (BRB) with the retinal pigment epithelium cells and endothelial cells of the retinal blood vessels.^[36–38]^ BRB breakdown in the central area results in swelling and fluid leakage leading to DME. Morphological changes and extracellular fluid accumulation alter the light path.^[39]^. Anti-VEGF treatment resolves the breakdown of the BRB and displacements of the photoreceptors result in the improvement of metamorphopsia.^[40]^ In our study, we showed improvement in both metamorphopsia and RS. These results indicate the improvement in other aspects of visual function beyond the VA. However, we evaluated metamorphopsia only with the subjective W/A test and not with observational tests such as the M-chart test, which can quantify the degree of metamorphopsia by assessing 20 degree of the visual field.^[28]^ Thus, it is not clear whether the degree of metamorphopsia improvement correlates with the RS improvement in DME patients with good vision. Further investigations are required about this.

There are some limitations to our study. First, our study has a small sample size. Second, although other previous studies reported a longer observation period (1 or 2 years) for anti-VEGF treatment for DME patients with a good VA,^[9,25]^ we showed only short-term results. In contrast to these studies, we treated patients with 3 consecutive monthly anti-VEGF injections. There is a possibility that the consecutive injection protocol at an early stage of treatment affects long-term functional outcomes. Therefore, the RS value evaluated by MP is a useful tool. It is possible that RS will be used as a predictor of visual and anatomical outcomes in the future. Third, we used different anti-VEGF agents and did not examine the differences between the agents. A previous study (DRCR.net-Protocol T) reported that both ranibizumab and aflibercept were more effective than bevacizumab, and there was a difference between ranibizumab and aflibercept for patients with a poor baseline VA.^[24]^ We could not compare the differences between the agents because of the small sample size. Further consideration is required for this. Fourth, MP is a time-consuming test compared with OCT because MP relies on patient cooperation. However, older instruments such as scanning laser ophthalmoscope (SLO; Rodenstock GmbH, Munich, Germany) or microperimetry-1 (MP-1, NIDEK, Gamagori, Japan) used in the earlier studies require longer time for examination compared with MAIA. In addition, newest microperimetry-3 (MP-3, NIDEK, Gamagori, Japan) can perform examination with much shorter time. Thus, technological improvements will resolve this problem in the future. Finally, our study included patients with a baseline decimal BCVA better than 0.6 (20/33), which is not necessarily a good BCVA as Protocol-V defined, although such patients were not included in previous RCTs. The baseline decimal VA better than 0.8 (20/25) needs to be considered in longitudinal observations.

In conclusion, our results show that MP is a good option for determining the efficacy of anti-VEGF treatment for DME patients with relatively good VA. We also found that anti-VEGF treatment is effective for metamorphopsia. In eyes with good initial vision, we need indices to show visual or anatomic improvement. Hence, MP and metamorphopsia qualitative tests are useful to monitor the effect of anti-VEGF treatment for tnem.

## Acknowledgments

The authors thank Professor Emeritus Duco Hamasaki of the Bascom Palmer Eye Institute of the University of Miami and Editage for critical discussion and final manuscript revisions.

## Author contributions

M.S. and M.K. designed the study. M.S. contributed to writing the manuscript. Y.W., R.M., and H.M. examined the patients. K.K. gave critical suggestions. All authors reviewed the manuscript.

**Conceptualization:** Masahiko Sugimoto, Mineo Kondo.

**Data curation:** Masahiko Sugimoto, Yasuko Wakamatsu, Ryohei Miyata, Hisashi Matsubara.

**Formal analysis:** Masahiko Sugimoto.

**Investigation:** Masahiko Sugimoto.

**Methodology:** Masahiko Sugimoto.

**Project administration:** Masahiko Sugimoto.

**Supervision:** Kumiko Kato, Mineo Kondo.

**Validation:** Masahiko Sugimoto.

**Writing – original draft:** Masahiko Sugimoto.

**Writing – review & editing:** Yasuko Wakamatsu, Ryohei Miyata, Kumiko Kato, Hisashi Matsubara, Mineo Kondo.
